# The Complex Energy Landscape of the Protein IscU

**DOI:** 10.1016/j.bpj.2015.07.045

**Published:** 2015-09-01

**Authors:** Jameson R. Bothe, Marco Tonelli, Ibrahim K. Ali, Ziqi Dai, Ronnie O. Frederick, William M. Westler, John L. Markley

**Affiliations:** 1 Department of Biochemistry, University of Wisconsin-Madison, Madison, Wisconsin; 2 National Magnetic Resonance Facility at Madison, University of Wisconsin-Madison, Madison, Wisconsin

## Abstract

IscU, the scaffold protein for iron-sulfur (Fe-S) cluster biosynthesis in *Escherichia coli*, traverses a complex energy landscape during Fe-S cluster synthesis and transfer. Our previous studies showed that IscU populates two interconverting conformational states: one structured (S) and one largely disordered (D). Both states appear to be functionally important because proteins involved in the assembly or transfer of Fe-S clusters have been shown to interact preferentially with either the S or D state of IscU. To characterize the complex structure-energy landscape of IscU, we employed NMR spectroscopy, small-angle x-ray scattering (SAXS), and differential scanning calorimetry. Results obtained for IscU at pH 8.0 show that its S state is maximally populated at 25°C and that heating or cooling converts the protein toward the D state. Results from NMR and DSC indicate that both the heat- and cold-induced S→D transitions are cooperative and two-state. Low-resolution structural information from NMR and SAXS suggests that the structures of the cold-induced and heat-induced D states are similar. Both states exhibit similar ^1^H-^15^N HSQC spectra and the same pattern of peptidyl-prolyl peptide bond configurations by NMR, and both appear to be similarly expanded compared with the S state based on analysis of SAXS data. Whereas in other proteins the cold-denatured states have been found to be slightly more compact than the heat-denatured states, these two states occupy similar volumes in IscU.

## Introduction

Although cold denaturation is a fundamental aspect of the protein free-energy landscape, general questions remain regarding its associated structures and energetics (1). The generally accepted model of cold denaturation involves a reduction of the hydrophobic effect such that the hydration of nonpolar groups becomes more favorable at low temperatures. One of the most puzzling aspects of cold unfolding is its equivalence to the heat-unfolding process. Previous reports have drawn contrasting conclusions regarding the cooperativity of the cold-unfolding process. From a structural standpoint, several studies have reported differences between heat- and cold-stabilized unfolded states; in particular, the cold-unfolded state is generally thought to be more compact. The biggest challenge in answering basic thermodynamic questions about cold denaturation is identifying proteins that undergo the process without the assistance of destabilizing effects such as the presence of alcohols or denaturants, confinement in micelles, or extreme pressure (1–10). Although these destabilizing effects have been crucial in allowing insights into cold-denatured states, it is difficult to fully decouple their effect on the protein-folding process from both an energetic and a structural standpoint. This is a key issue because hydration and the hydrophobic effect are generally accepted to be crucial to cold-stabilized unfolded conformations. Recently, thermodynamic and structural studies have been carried out on a few proteins that were found to undergo cold denaturation without the addition of denaturants (11–14). For example, extensive studies of yeast frataxin (Yfh1) (12,15–17) and a C-terminal domain of a variant of ribosomal protein L9 (CTL9) (11,18,19) have provided detailed insights into cold denaturation. In general, the cold-unfolded state for both proteins appeared to be more compact than the heat-unfolded state, and the cold-unfolding process was found to be cooperative. Here, we used NMR spectroscopy, differential scanning calorimetry (DSC), and small-angle x-ray scattering (SAXS) to characterize the structure and energetics associated with the heat- and cold-induced order-to-disorder transition in *Escherichia coli* IscU, the highly conserved scaffold protein for iron-sulfur (Fe-S) cluster biosynthesis. Our results show that the cold- and heat-induced order-to-disorder transitions in IscU are cooperative, and the heat- and cold-stabilized conformations are similarly expanded.

## Materials and Methods

### Sample preparation

A pE-SUMO plasmid (Lifesensors) containing either the *E. coli* IscU gene or its variants, IscU(D39A) and IscU(P101A), was transformed into BL21 RILP(DE3) cells (Stratagene). Unlabeled protein was produced by growing the cells for 48 h at 12°C in an autoinduction medium (20). The autoinduction protocol was similar to that used recently to produce *E. coli* IscX (21), except for a revised temperature and growth time. IscU samples labeled with ^13^C and ^15^N were prepared by growing cells in M9 minimal media containing [U-^13^C]-glucose and ^15^NH_4_Cl. Protein production was induced when OD_600_ reached ∼1.0 by the addition of isopropyl *β*-D-1 thiogalactoside to a concentration of 0.2 mM. After induction, the cells were grown for 48 h at 12°C. After protein expression, the cells were pelleted and placed into a −80°C freezer pending protein purification.

Frozen cells were suspended in buffer (20 mM TRIS pH 8.0, 500 mM NaCl, 0.3 mM tris(2-carboxyethyl)phosphine (TCEP), 5 mM imidazole), lysed by sonication, and centrifuged. The supernatant was loaded onto a column containing Qiagen Nickel-NTA Superflow resin. Protein was eluted from the column with imidazole buffer (20 mM TRIS pH 8.0, 500 mM NaCl, 0.3 mM TCEP, 250 mM imidazole), and fractions containing the SUMO-IscU fusion were pooled. The eluted fractions were dialyzed overnight into SUMO protease cleavage buffer (20 mM TRIS pH 8.0, 100 mM NaCl, 0.3 mM TCEP) at 4°C in the presence of SUMO protease. Next, the cleaved protein was loaded onto an IMAC column, which bound the His-tagged SUMO and any uncleaved fusion protein. Column flow-through and washes containing IscU were pooled, and product-containing fractions were concentrated with spin concentrators (Sartorius Stedim Vivaspin 20, 5 kDa MWCO PES) and further purified by gel filtration (Hi-Load 26/60 Superdex 200 prep grade).

Unless specified otherwise, protein samples were exchanged into 50 mM TRIS pH 8.0, 150 mM NaCl, 0.1 mM EDTA, 5 mM dithiothreitol (DTT), or into 50 mM HEPES pH 8.0, 150 mM NaCl, 0.1 mM EDTA, 5 mM DTT. The reductant used for SAXS studies was either DTT or TCEP as indicated. Because both the S ⇄ D equilibrium of IscU (22) and the p*K*_a_ of TRIS depend on temperature, we compared results obtained from using TRIS or HEPES buffer; however, we observed no difference.

### NMR spectroscopy

The buffers used for NMR experiments were as described above but with the inclusion of 10% D_2_O (for the frequency lock) and 50 *μ*M of 4,4-dimethyl-4-silapentane-1-sulfonic acid (as the internal chemical-shift reference). NMR spectra were recorded at the National Magnetic Resonance Facility at Madison (University of Wisconsin-Madison) on a 600 MHz Varian VNMRS spectrometer or a 600 MHz Bruker Avance III spectrometer, each equipped with *z*-gradient cryogenic probes. For temperature-dependent studies, samples were allowed to equilibrate for a minimum of 15 min before data acquisition. Raw NMR data were processed with NMRPipe (23), and peak intensities were measured by the fast maximum-likelihood reconstruction (FMLR) method (24).

### DSC

A Microcal VP differential scanning calorimeter was used for collection of DSC data. Protein samples were extensively dialyzed (≥24 h) before DSC data were recorded. DSC thermograms for IscU and IscU(D39A) were recorded as follows: samples were cooled to 1°C, held at 1°C for 15 min, and then warmed to 70°C at a rate of 1°C/min. This temperature scheme was then cycled several times. Thermograms for IscU and IscU(D39A) were reversible with a loss of signal of ∼5–10% per temperature cycle.

### SAXS

Protein samples for SAXS were dialyzed extensively before data collection. Anaerobic samples were prepared by dialyzing IscU or IscU(D39A) in degassed buffer for >12 h in an anaerobic chamber (Coy Laboratories) with O_2_ < 1 ppm and 3−6% H_2_. Anaerobic samples were placed in an airtight SAXS cell within the glove box. The Bruker Nanostar benchtop SAXS system at the National Magnetic Resonance Facility at Madison was used for data collection. The Bruker Nanostar system was equipped with a rotating anode (Cu) Turbo X-Ray Source and a Vantec-2000 (2048 × 2048 pixel) detector (Bruker AXS). The sample-to-detector distance was set at ∼67 cm, allowing for a detection range of 0.012 Å^−1^ > *q* > 0.383 Å^−1^. SAXS data were collected from IscU and IscU(D39A) samples at protein concentrations ranging from 1.5 to 6.0 mg/mL. Sample and buffer scattering data were collected over periods of 2–4 h, with frames recorded every hour. For temperature-dependent studies, the temperature was held for a minimum of 15 min before data were recorded, to allow for equilibration. The SAXS data sets were averaged and converted to 1D scattering profiles for further analysis. The ATSAS (25) software suite was used to carry out buffer subtraction and process the SAXS data. The radius of gyration (*R*_g_) was determined by using the Guinier approximation in the *q* range, such that *q*_max_ ≤ *R*_g_ ≤ 1.0.

### Minimal ensemble analysis of SAXS data

The minimal ensemble search (MES) algorithm (26) was used to carry out an ensemble analysis of temperature-dependent SAXS data from IscU. An ensemble of 1000 structures composed of fully folded to fully extended structures was used for the selection pool. Other structural models were obtained from a 100 ns molecular-dynamics trajectory generated with AMBER 12 (27), using the structured conformation of IscU as the starting structure. The simulation was carried out using a generalized Born implicit solvent model (28) along with SHAKE (29) and Langevin dynamics with a collision frequency of 2 ps^−1^ (30). A simulation temperature of 373 K was found to yield unfolded structures. The structure pool was augmented by the ensemble of 20 NMR conformers representing the solution structure of the S state of IscU (8).

We used MES to select one- and two-state ensembles that best fit the experimental SAXS data. At all temperatures, the two-state ensemble achieved a slightly better fit (X value) to the experimental SAXS data. Fits were also validated with the recently proposed $X_{\text{free}}$ statistic. The ScÅtter software package (http://www.bioisis.net/tutorial) was used to calculate $X_{\text{free}}$ with 5000 selection rounds.

## Results and Discussion

**Figure 1:**
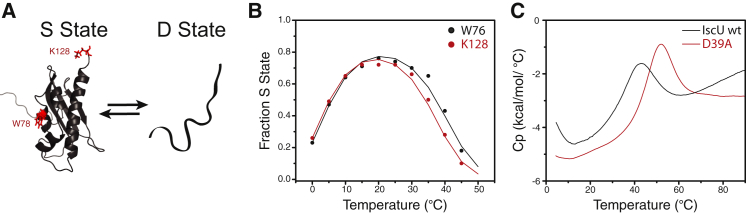
Energetic landscape of IscU. (*A*) IscU exists in equilibrium between a structured (S) state (PDB ID: 24LX) (8) and a disordered (D) state. (*B*) Temperature dependence of the fraction of the protein molecules in the S state, ([S]/([S]+[D])), as determined from the relative intensities of NMR signals assigned to W76 (*black circles*) and K128 (*red circles*) in the two conformational states. The curves resulted from fitting each data set to the Gibbs-Helmholtz equation (Table 1). (*C*) DSC thermograms of IscU (*black*) and D39A IscU (*red*).

**Figure 2:**
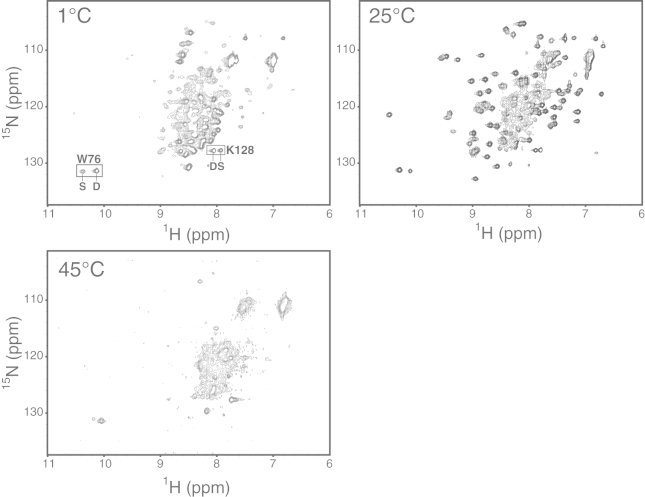
^1^H-^15^N HSQC spectra of IscU collected at the temperatures indicated, showing the collapse of the spectrum upon cooling or warming. Boxes highlight the resonances used to determine the S- and D-state populations for thermodynamic analysis (Fig. 1*B*).

Under physiological conditions, IscU populates two slowly interchanging conformational states: one (S) is structured and the other (D) is dynamically disordered (Fig. 1*A*). The S and D states interconvert with *k*_ex_ ∼1 s^−1^ at 25°C, i.e., at a rate slow enough for separate NMR signals to be observed for the two states (31). Earlier NMR studies showed that the D state became favored at higher or lower temperatures (32), and this was confirmed more recently by circular dichroism (33). We previously determined the solution structure of the S state of IscU, which consists of four *α*-helices and three antiparallel *β*-strands (8). The D state yields a poorly dispersed ^1^H-^15^N heteronuclear single quantum coherence (HSQC) spectrum indicative of dynamic disorder (Fig. 2), and the lack of secondary chemical shifts indicates that the D state contains minimal secondary structure (34).

Prior 2D ^1^H-^15^N NMR studies of ^15^N-labeled IscU assigned separate signals from Trp-76 (W76) ^1^H^*ε*1_15^N^*ε*1^ and Lys-128 (K128) ^1^H-^15^N to the S and D states (8,35). These signals are well resolved at temperatures between 1°C and 45°C (Fig. 2), and their relative intensities in the two states as determined by FMLR (24) served as excellent probes for the relative populations of each state. This analysis of IscU at pH 8.0 showed that the S state is maximally populated (75%) at ∼25°C, but raising or lowering the temperature shifts the equilibrium toward the D state (Figs. 1*B* and 2). The probes located in two very different locations in the protein yielded similar temperature-dependent populations, as expected for a global conformational change (Fig. 1*B*). The slight divergence at high temperatures likely stems from differential exchange rates of the protons with bulk water. We note that the quality of the ^1^H-^15^N HSQC spectra diminished with temperatures at or above 45°C, likely as the result of accelerated proton exchange with solvent. The temperature-dependent populations provided an excellent fit to the Gibbs-Helmholtz equation (Fig. 1*B*; Table 1) (36). Thus, thermodynamically, the S-to-D transition in IscU can be described as a two-state process that is energetically symmetric at high and low temperatures.

The DSC thermogram for IscU exhibited two clear conformational transitions (Fig. 1*C*), with upticks in the measured heat capacity near the hot and cold transition temperatures observed by NMR (Fig. 1*B*). Cooperative transitions in proteins generally display significant heat absorption at the unfolding/melting temperature due to a sharp change in the populations of folded and unfolded states, with different enthalpies near the melting temperature (37). By contrast, noncooperative transitions, such as those observed for molten-globule states, result in linear changes in the observed partial heat capacity (Cp) as a function of temperature (38–40). Thus, the thermograms for the heat- and cold-induced transitions of IscU indicate that an extensive network of structurally stabilizing interactions is lost upon formation of the D state. The low- and high-temperature limbs are consistent with the NMR-based thermodynamic analysis, which indicated cooperative heat- and cold-induced order-to-disorder transitions with ΔH positive and negative on opposite sides of the free-energy surface. As a control, we collected DSC data on the D39A variant of IscU, whose S state is much more stable than that of wild-type IscU (8). The DSC thermogram for IscU (D39A) exhibited a single large unfolding transition at ∼50°C, as expected for a classic globular protein (Fig. 1*C*).

We next sought to gain insights into the energetic landscape of IscU by probing structural aspects of its heat- and cold-stabilized disordered conformations. A previous study showed that all four prolines of IscU have *trans* peptidyl-prolyl peptide bonds in the S state, whereas two of the four (P14 and P101) become *cis* in the heat-induced D state (41). One can determine the *cis*/*trans* conformations of peptidyl-prolyl bonds by analyzing proline side-chain chemical shifts. Specifically, the chemical-shift difference between a proline’s *δ*^13^C^*β*^ and *δ*^13^C^*γ*^ signals is ∼5 or ∼10 ppm when its configuration is *trans* or *cis*, respectively (42–44). 3D HCCH TOCSY data from a sample of IscU containing [U-^13^C]-proline at a temperature of 5°C showed the same pattern of (*δ*^13^C^*β*^ − *δ*^13^C^*γ*^) chemical-shift differences observed at 45°C (41) (Fig. S1 in the Supporting Material).

To expand our focus from local to global structural information, we collected SAXS data, which provide low-resolution information about molecular shapes and offer a powerful means of studying disordered protein conformations (45). First, we prepared samples under buffer conditions identical to those previously used to study the structure and dynamics of IscU by NMR (8,31,34,35,41), and collected SAXS data on a Bruker Nanostar system equipped with a variable-temperature sample stage (Table S1). Surprisingly, we observed no change in the *R*_g_ of IscU upon a decrease in temperature (Fig. S2 A). However, as expected, the *R*_g_ of IscU increased significantly from ∼23 Å to ∼30 Å upon heating. To further characterize this unexpected behavior, we determined the molecular mass of IscU using the new SAXS invariant parameter, *V*_c_ (46). The *V*_c_ method has been shown to be applicable for both compact and disordered molecules. By contrast, the commonly utilized zero angle scattering *I*(0) method (47) is most accurate when the electron density of the molecular species of interest resembles those of the molecular mass standards usually employed (globular proteins). The *V*_c_ approach yielded an average mass of 28.7 kDa over the entire temperature range (Fig. S2 B), a value very close to the mass of dimeric IscU (27.7 kDa). It is likely that the size of the dimeric form of IscU was masking differences between the cold- and heat-stabilized S and D states.

Subsequent SAXS studies of a protein variant, IscU(P101A), under the same buffer conditions indicated that the protein converted slowly over time from a monomer to a dimer (Fig. S3). Over the course of the variable-temperature study, the observed molecular weight shifted from that expected for a monomer to that expected for a dimer. This result led us to suspect that dimerization was occurring by formation of one or more intermolecular disulfide bridges. SAXS sample preparation requires extensive dialysis (>12 h), and the process may have led to oxidation of the reductant (DTT) in the wild-type IscU sample (Fig. S2).

**Figure 3:**
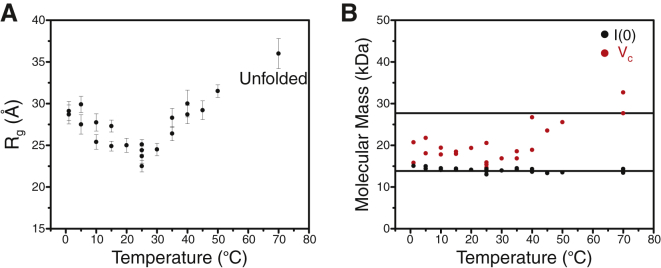
Temperature-dependent SAXS studies of monomeric IscU prepared with 10 mM TCEP in an anaerobic chamber. (*A*) *R*_g_ values calculated from SAXS data collected at different temperatures. (*B*) Molecular mass of IscU as determined from SAXS data by the *I*(0) (*black*) and *V*_c_ (*red*) methods. The lines indicate the molecular masses of monomeric (13.8 kDa) and dimeric (27.7 kDa) IscU.

To ensure that the cysteines of IscU remained reduced, we prepared samples in an anaerobic chamber. IscU was dialyzed extensively against degassed buffer containing 10 mM TCEP, and samples were placed in a sealed SAXS sample cell. IscU prepared under these conditions at 3.0 or 6.0 mg/mL appeared to be monomeric over the temperature range studied (Fig. 3). The SAXS results revealed large increases in the *R*_g_ upon cooling and heating (Fig. 3*A*), consistent with the disorder implied from the collapsed NMR spectra at high and low temperatures.

**Figure 4:**
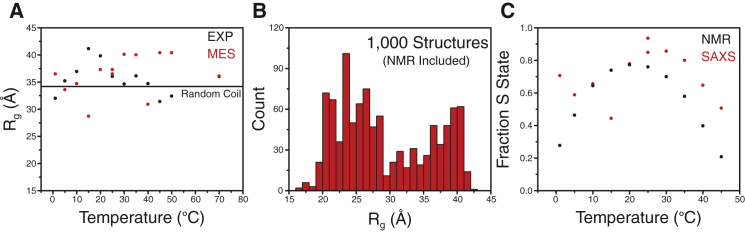
Low-resolution structural analysis of IscU by SAXS. (*A*) D-state *R*_g_ values calculated from experimental SAXS data using Eq. 1 (*black*) and from disordered structures selected from a two-state MES (*red*). (*B*) *R*_g_ values for members of the ensemble of structures used for the MES analysis. (*C*) Fractional S-state population derived from the conformers selected by the two-state MES fit (*red*) compared with that determined by NMR (*black*).

Assuming a two-state model for the S ⇄ D equilibrium between 0°C and 50°C, the experimentally measured *R*_g_ (*R*_gEXP_) is given by Eq. 1,

(1) $$R_{gEXP}^{2}=p_{s}R_{gS}^{2}+p_{D}R_{gD}^{2},$$

where *p*_S_ and *p*_D_ are the populations of the two states, and *R*_gS_ and *R*_gD_ are the *R*_g_ of the two states (48). We assumed an S-state *R*_g_ of 18.9 Å over this temperature range, as derived from the NMR structural ensemble (8). As a control for the S state, we carried out temperature-dependent studies of the structured variant IscU(D39A) (8). Its *R*_g_ did not change between 0°C and 25°C, and only increased at high temperatures (Fig. S4). The calculated D-state *R*_g_ of IscU remained nearly constant (*R*_gD_ = 35.5 ± 2.9 Å) over the entire temperature range (Fig. 4*A*). Interestingly, the *R*_g_ predicted for IscU in a random chain configuration is 34.2 Å (49), and this similarity to the experimental values obtained at high and low temperatures suggests that both the heat- and cold-induced D states of IscU are highly extended with very little residual structure.

**Figure 5:**
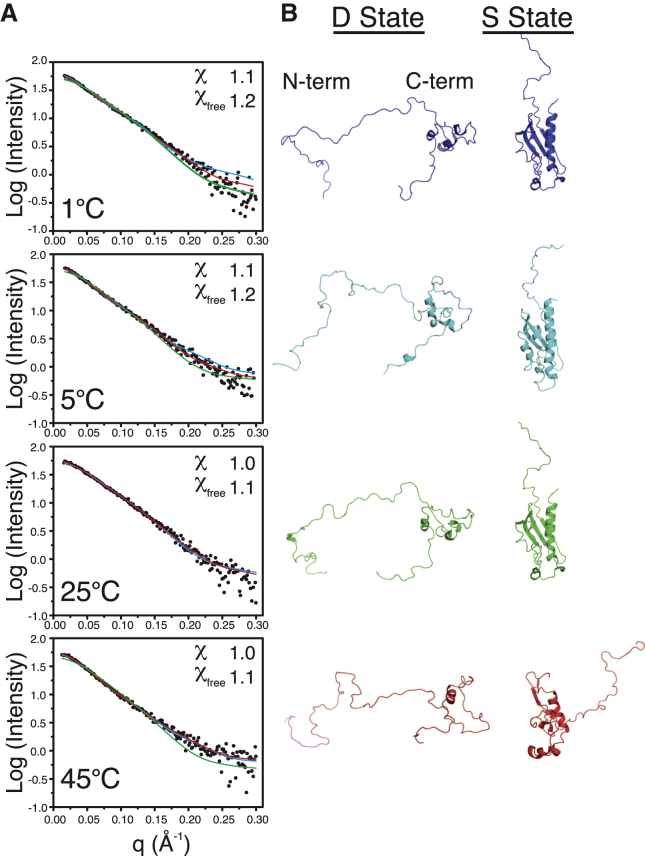
MES results for temperature-dependent IscU SAXS data. (*A*) Experimental SAXS data (*circles*) collected at different temperatures are compared with fits to MES against one structure (*blue*) and two structures (*red*). X and $X_{\text{free}}$ statistics are shown for the two-structure MES fits. For reference, the fit (*green*) to the single structure (*R*_g_ of 22 Å) selected for IscU at 25°C is shown for 1°C, 5°C, and 45°C. (*B*) Structures selected in the two-state MES fits to the experimental SAXS data at different temperatures.

We utilized an ensemble-based analysis of the SAXS data to gain deeper insight into the conformation of IscU’s D state. The MES approach uses an advanced genetic algorithm to select minimal ensembles of conformers from a structure pool that best agree with experimental SAXS data (26). The conformer pool we used for MES comprised the NMR ensemble of conformers representing the S state of IscU (8) augmented by 1000 structures derived from molecular-dynamics simulations, varying from fully folded to fully extended (Fig. 4*B*). Fig. 5*A* shows the best fit upon MES selection for single- and two-state models. At each temperature the fit was improved by considering two conformations, and the resulting two-state fits yielded X and $X_{\text{free}}$ values of ∼1 (45). Increasing the ensemble size by up to four conformers did not result in additional improvement of the fit beyond the two-state model for all temperatures. Although the SAXS data can be fit to a single model with varying quality (∼1 ≤ $X_{\text{free}}$ ≤ 1.6), our NMR results clearly indicate that two states are present. This highlights the limitation of using SAXS data alone, since SAXS is unable to independently discern the difference between a single state and multiple states that satisfy the experimental IscU data. Given our knowledge from NMR that IscU exists in an S and a D state, we sought to determine what combination of the structural models (S and D) would be consistent with the SAXS data. Interestingly, when we carried out a two-state MES fit, the populations of the selected S and D conformers generally trended consistently with those observed by NMR (Fig. 4*C*). The *R*_g_ values of the disordered conformations selected by MES were also consistent with those calculated from Eq. 1 (Fig. 4*A*). Further, the structural models selected were consistent with the NMR observation of a folded and disordered conformation (Fig. 5*B*). Overall, our SAXS data combined with NMR suggest that the cold- and heat-induced D states of IscU are both highly extended and are nearly in a random-coil conformation. Residual structure in the D state may exist in regions around its two *cis* prolines (P14 and P101) and some secondary structure in the C-terminus.

## Conclusions

We utilized a hybrid approach employing NMR, DSC, and SAXS to characterize the complex energetic landscape of IscU. The low-resolution structural insight afforded by hybrid NMR/SAXS analysis suggests that the energetic landscape of IscU’s S ⇄ D equilibrium is symmetric both energetically and structurally. Similarly to Yfh1 (12,15) and a CTL9 mutant (11,18,19), IscU underwent a two-state cooperative S→D conformational transition upon an increase or decrease in temperature. However, unlike Yfh1 and the CTL9 mutant, which exhibited more compact cold-denatured states, the heat- and cold-induced D states of IscU appeared to be equally extended. In addition, the *R*_g_ of the cold-induced D state of IscU did not increase with decreasing temperature as was observed for the cold-denatured state of Yfh1 (17). Such protein-specific differences highlight the need to develop a larger canon of model systems for studying the process of cold denaturation so that its energetic and structural aspects can be more fully understood.

## Author Contributions

J.L.M., J.R.B., and Z.D. designed the research. M.T., Z.D., I.K.A., and J.R.B. collected and interpreted the NMR data. J.R.B. collected and interpreted the DSC and SAXS data. R.O.F. prepared protein samples. W.M.W. carried out the molecular-dynamics simulations. All authors contributed to drafting and approving the manuscript.

## Figures and Tables

**Table 1:** Fits of Temperature-Dependent NMR Data to the Gibbs-Helmholtz Equation with a Reference Temperature of 25°C

NMR Probe	Δ*H* (kcal ⋅ mol^−1^)	Δ*S* (cal ⋅ mol^−1^ ⋅ K^−1^)	Δ*Cp* (kcal ⋅ mol^−1^ ⋅ K^−1^)
W76	6.52 ± 0.7	19.6 ± 2.3	1.71 ± 0.1
K128	10.3 ± 0.4	32.8 ± 1.4	1.82 ± 0.1

NMR data from Fig. 1*B*. Gibbs-Helmholtz equation: Δ*G*(*T*) = Δ*H*(*T*_ref_) − *T*Δ*S*(*T*_ref_) + Δ*Cp*[(*T* − *T*_ref_) − *T* ln(*T*/*T*_ref_)], where *T*_ref_ is the reference temperature.

## Supporting Citations

References (8,41) appear in the Supporting Material.

## Supporting Material

Document S1. Four figures and one table

Document S2. Article plus Supporting Material
