# Supplementary material for: The Complex Energy Landscape of the Protein IscU
Source: Biophys J. 2015 Sep 1;109(5):1019–25. doi: 10.1016/j.bpj.2015.07.045 (PMC4564936; doi:10.1016/j.bpj.2015.07.045)
Supplement: Document S1. Four figures and one table [file mmc1.pdf]

# Complex energy landscape of the protein IscU

Jameson R. Bothe[a,b], Marco Tonelli[b], Ibrahim Ali[a], Ziqi Dai[a,b], Ronnie O. Frederick[c], William M. Westler[b], and John L. Markley[a,b,c]*

[a]Department of Biochemistry, [b]National Magnetic Resonance Facility at Madison, and [c]Mitochondrial Protein Partnership, Center for Eukaryotic Structural Genomics University of Wisconsin, 433 Babcock Drive, Madison, WI 53706

*To whom correspondence should be addressed.

Phone: (608) 263-9349. Fax: (608) 262-3759.

Email: jmarkley@wisc.edu.

## Supporting Material

**TABLE S1.** Summary of the SAXS data.

| | |
|---|---|
| Sample | IscU |
| Data-collection parameters | |
| Instrument | Bruker Nanostar |
| Beam geometry | 2 pinholes (500 μm) |
| Wavelength (Å) | 1.5418 Å |
| $q$ range (Å$^{-1}$) | 0.012 – 0.384 |
| Exposure time (h) | 2-4 |
| Concentration range (mgml$^{-1}$) | 1.5-6.0 |
| Temperature (K) | 274 - 343 |
| Molecular-mass determination | |
| Molecular mass [I(0)] (kDa) | Sample Prep Dependent |
| Molecular mass [$V_c$] (kDa) | Sample Prep Dependent |
| Calculated molecular mass from sequence (kDa) | 13.9 |
| Software Employed | |
| Primary data reduction | SAXS (Bruker) |
| Data processing | PRIMUS |
| Computation of model intensities | FoXS |
| Ensemble fitting | MES |
| Three-dimensional graphics representations | Pymol |

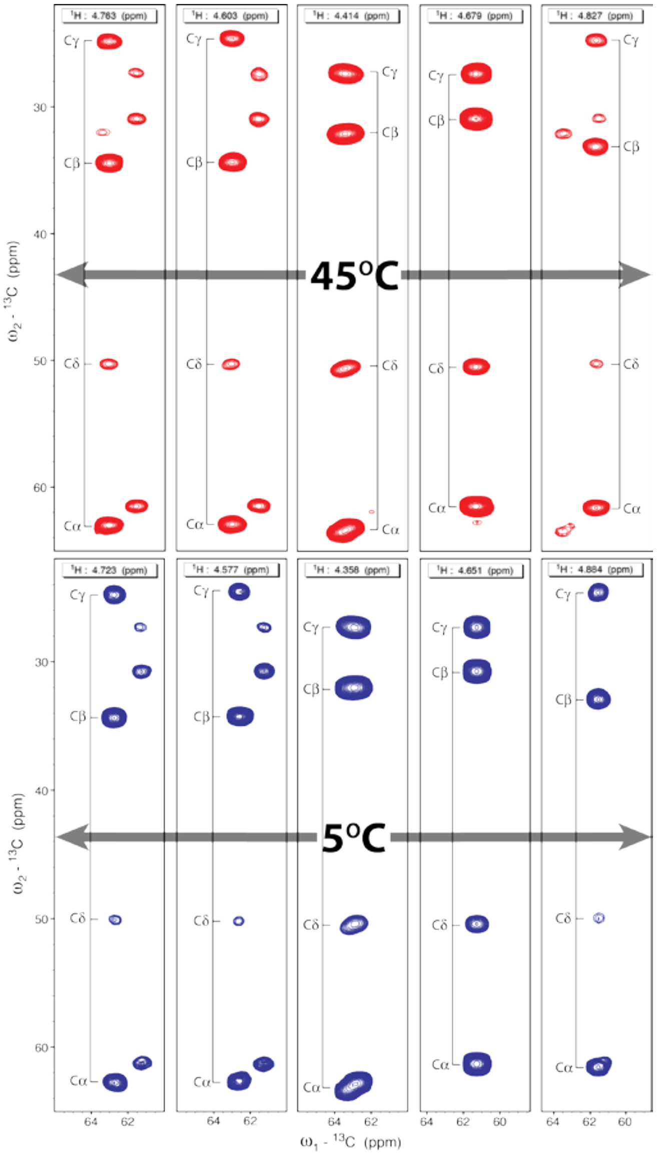


**FIGURE S1** Slices through 3D HCCH-TOSCY spectra at the $^{1}$H frequencies shown at the top of each slice. Proline residues of IscU in both its heat- and cold-induced disordered states adopt a similar mixture of *cis* and *trans* peptide bonds as exhibited by the chemical shift difference between the $^{13}C^{\beta}$ and $^{13}C^{\gamma}$ signals. Strips 1, 2, and 5 have $(\delta^{13}C^{\beta} - \delta^{13}C^{\gamma}) \approx 10$ ppm indicative of a *cis* peptidyl prolyl peptide bond, and strips 3 and 4 have $(\delta^{13}C^{\beta} - \delta^{13}C^{\gamma}) \approx 5$ ppm indicative of a *trans* peptidyl prolyl peptide bond. The strips at 45 °C were assigned previously (1) as follows: strips 1 and 2 to two conformations of Pro14; strip 3 to Pro 35, strip 4 to Pro100, and strip 5 to Pro101.

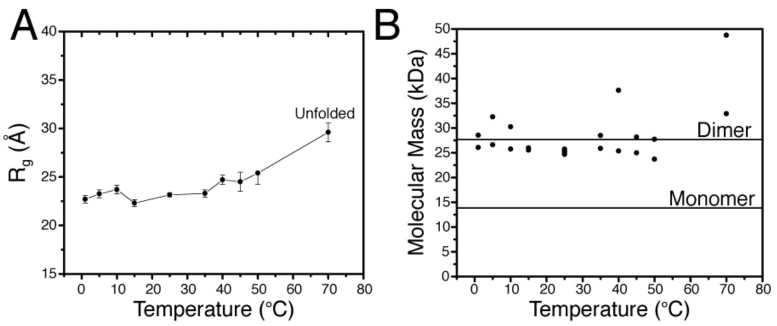


**FIGURE S2** Temperature-dependent SAXS studies of IscU prepared by dialyzing into a buffer with 5 mM DTT. (A) Radius of gyration ($R_g$) calculated from SAXS data collected at different temperatures. (B) Molecular mass of IscU determined from the SAXS data by the $V_c$ method. The lines indicate the molecular masses of monomeric (13.8 kDa) and dimeric (27.7 kDa) IscU.

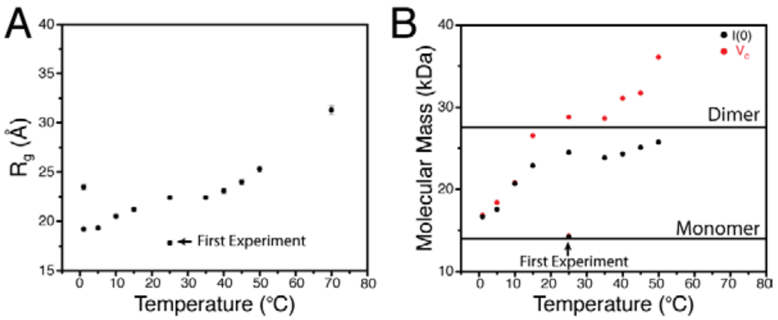


**FIGURE S3** Evidence for the dimerization of IscU over time as DTT oxidizes. (A) Radius of gyration ($R_g$) calculated from SAXS data collected at different temperatures. The first experiment was recorded at 25 °C. The sample was then cooled to 1°C and the temperature was raised in steps to 70 °C. (B) Molecular mass of IscU determined from SAXS data by the *I*(0) (red) and $V_c$ (black) methods. The lines indicate the molecular masses of monomeric (13.8 kDa) and dimeric (27.7 kDa) IscU.

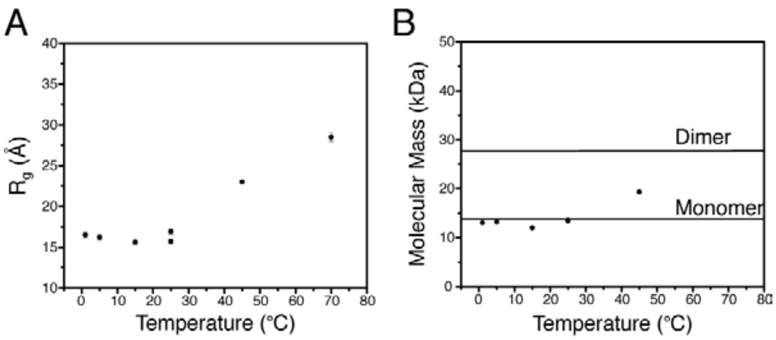


**FIGURE S4** Temperature-dependent SAXS data for IscU(D39A), which has a much more stable S-state than wild-type IscU (2). (A) experimentally observed $R_g$. (B) molecular mass of IscU(D39A) determined by the $V_c$ method. The lines indicate the molecular masses of monomeric (13.8 kDa) and dimeric (27.7 kDa) IscU(D39A). Unlike IscU, the $R_g$ of IscU(D39A) does not increase significantly upon decreasing the temperature. From NMR studies, the S-state of IscU(D39A) is populated at ~95%. IscU(D39A) transitions to the D-state at 50 °C and unfolds completely at 70 °C.

## References

1. Dai, Z., M. Tonelli, and J. L. Markley. 2012. Metamorphic Protein IscU Changes Conformation by *cis-trans* Isomerizations of Two Peptidyl-Prolyl Peptide Bonds. *Biochemistry* 51:9595-9602.

2. Kim, J. H., M. Tonelli, T. Kim, and J. L. Markley. 2012. Three-Dimensional Structure and Determinants of Stability of the Iron-Sulfur Cluster Scaffold Protein IscU from *Escherichia coli*. *Biochemistry* 51:5557-5563.
