# Supplementary material for: The Complex Energy Landscape of the Protein IscU
Source: Biophys J. 2015 Sep 1;109(5):1019–25. doi: 10.1016/j.bpj.2015.07.045 (PMC4564936; doi:10.1016/j.bpj.2015.07.045)
Supplement: Document S2. Article plus Supporting Material [file mmc2.pdf]

Biophysical Journal Volume 109 September 2015 1019–1025

Article

The Complex Energy Landscape of the Protein IscU

Jameson R. Bothe,[1,2] Marco Tonelli,[2] Ibrahim K. Ali,[1] Ziqi Dai,[1,2] Ronnie O. Frederick,[1,2] William M. Westler,[2] and John L. Markley[1,2,*]
[1]Department of Biochemistry and [2]National Magnetic Resonance Facility at Madison, University of Wisconsin-Madison, Madison, Wisconsin

ABSTRACT IscU, the scaffold protein for iron-sulfur (Fe-S) cluster biosynthesis in *Escherichia coli*, traverses a complex energy landscape during Fe-S cluster synthesis and transfer. Our previous studies showed that IscU populates two interconverting conformational states: one structured (S) and one largely disordered (D). Both states appear to be functionally important because proteins involved in the assembly or transfer of Fe-S clusters have been shown to interact preferentially with either the S or D state of IscU. To characterize the complex structure-energy landscape of IscU, we employed NMR spectroscopy, small-angle x-ray scattering (SAXS), and differential scanning calorimetry. Results obtained for IscU at pH 8.0 show that its S state is maximally populated at 25°C and that heating or cooling converts the protein toward the D state. Results from NMR and DSC indicate that both the heat- and cold-induced S→D transitions are cooperative and two-state. Low-resolution structural information from NMR and SAXS suggests that the structures of the cold-induced and heat-induced D states are similar. Both states exhibit similar ^{1}H-^{15}N HSQC spectra and the same pattern of peptidyl-prolyl peptide bond configurations by NMR, and both appear to be similarly expanded compared with the S state based on analysis of SAXS data. Whereas in other proteins the cold-denatured states have been found to be slightly more compact than the heat-denatured states, these two states occupy similar volumes in IscU.

INTRODUCTION

Although cold denaturation is a fundamental aspect of the protein free-energy landscape, general questions remain regarding its associated structures and energetics (1). The generally accepted model of cold denaturation involves a reduction of the hydrophobic effect such that the hydration of nonpolar groups becomes more favorable at low temperatures. One of the most puzzling aspects of cold unfolding is its equivalence to the heat-unfolding process. Previous reports have drawn contrasting conclusions regarding the cooperativity of the cold-unfolding process. From a structural standpoint, several studies have reported differences between heat- and cold-stabilized unfolded states; in particular, the cold-unfolded state is generally thought to be more compact. The biggest challenge in answering basic thermodynamic questions about cold denaturation is identifying proteins that undergo the process without the assistance of destabilizing effects such as the presence of alcohols or denaturants, confinement in micelles, or extreme pressure (1–10). Although these destabilizing effects have been crucial in allowing insights into cold-denatured states, it is difficult to fully decouple their effect on the protein-folding process from both an energetic and a structural standpoint. This is a key issue because hydration and the hydrophobic effect are generally accepted to be crucial to cold-stabilized unfolded conformations. Recently, thermodynamic and structural studies have been carried out on a few proteins that were found to undergo cold denaturation without the addition of denaturants (11–14). For example, extensive studies of yeast frataxin (Yfh1) (12,15–17) and a C-terminal domain of a variant of ribosomal protein L9 (CTL9) (11,18,19) have provided detailed insights into cold denaturation. In general, the cold-unfolded state for both proteins appeared to be more compact than the heat-unfolded state, and the cold-unfolding process was found to be cooperative. Here, we used NMR spectroscopy, differential scanning calorimetry (DSC), and small-angle x-ray scattering (SAXS) to characterize the structure and energetics associated with the heat- and cold-induced order-to-disorder transition in *Escherichia coli* IscU, the highly conserved scaffold protein for iron-sulfur (Fe-S) cluster biosynthesis. Our results show that the cold- and heat-induced order-to-disorder transitions in IscU are cooperative, and the heat- and cold-stabilized conformations are similarly expanded.

MATERIALS AND METHODS

Sample preparation

A pE-SUMO plasmid (Lifesensors) containing either the *E. coli* IscU gene or its variants, IscU(D39A) and IscU(P101A), was transformed into BL21 RILP(DE3) cells (Stratagene). Unlabeled protein was produced by growing the cells for 48 h at 12°C in an autoinduction medium (20). The autoinduction protocol was similar to that used recently to produce *E. coli* IscX (21), except for a revised temperature and growth time. IscU samples labeled

Submitted April 6, 2015, and accepted for publication July 28, 2015.
*Correspondence: jmarkley@wisc.edu
This is an open access article under the CC BY license (http://creativecommons.org/licenses/by/4.0/).
Editor: Jeff Peng.
© 2015 The Authors
0006-3495/15/09/1019/7
CrossMark
http://dx.doi.org/10.1016/j.bpj.2015.07.045

with ^{13}C and ^{15}N were prepared by growing cells in M9 minimal media containing [U-^{13}C]-glucose and $^{15}NH_4Cl$. Protein production was induced when OD_{600} reached ~1.0 by the addition of isopropyl β-D-1 thiogalactoside to a concentration of 0.2 mM. After induction, the cells were grown for 48 h at 12°C. After protein expression, the cells were pelleted and placed into a −80°C freezer pending protein purification.

Frozen cells were suspended in buffer (20 mM TRIS pH 8.0, 500 mM NaCl, 0.3 mM tris(2-carboxyethyl)phosphine (TCEP), 5 mM imidazole), lysed by sonication, and centrifuged. The supernatant was loaded onto a column containing Qiagen Nickel-NTA Superflow resin. Protein was eluted from the column with imidazole buffer (20 mM TRIS pH 8.0, 500 mM NaCl, 0.3 mM TCEP, 250 mM imidazole), and fractions containing the SUMO-IscU fusion were pooled. The eluted fractions were dialyzed overnight into SUMO protease cleavage buffer (20 mM TRIS pH 8.0, 100 mM NaCl, 0.3 mM TCEP) at 4°C in the presence of SUMO protease. Next, the cleaved protein was loaded onto an IMAC column, which bound the His-tagged SUMO and any uncleaved fusion protein. Column flow-through and washes containing IscU were pooled, and product-containing fractions were concentrated with spin concentrators (Sartorius Stedim Vivaspin 20, 5 kDa MWCO PES) and further purified by gel filtration (Hi-Load 26/60 Superdex 200 prep grade).

Unless specified otherwise, protein samples were exchanged into 50 mM TRIS pH 8.0, 150 mM NaCl, 0.1 mM EDTA, 5 mM dithiothreitol (DTT), or into 50 mM HEPES pH 8.0, 150 mM NaCl, 0.1 mM EDTA, 5 mM DTT. The reductant used for SAXS studies was either DTT or TCEP as indicated. Because both the S $\rightleftarrows$ D equilibrium of IscU (22) and the pK_a of TRIS depend on temperature, we compared results obtained from using TRIS or HEPES buffer; however, we observed no difference.

NMR spectroscopy

The buffers used for NMR experiments were as described above but with the inclusion of 10% D_2O (for the frequency lock) and 50 μM of 4,4-dimethyl-4-silapentane-1-sulfonic acid (as the internal chemical-shift reference). NMR spectra were recorded at the National Magnetic Resonance Facility at Madison (University of Wisconsin-Madison) on a 600 MHz Varian VNMRS spectrometer or a 600 MHz Bruker Avance III spectrometer, each equipped with z-gradient cryogenic probes. For temperature-dependent studies, samples were allowed to equilibrate for a minimum of 15 min before data acquisition. Raw NMR data were processed with NMRPipe (23), and peak intensities were measured by the fast maximum-likelihood reconstruction (FMLR) method (24).

DSC

A Microcal VP differential scanning calorimeter was used for collection of DSC data. Protein samples were extensively dialyzed ($\geq$24 h) before DSC data were recorded. DSC thermograms for IscU and IscU(D39A) were recorded as follows: samples were cooled to 1°C, held at 1°C for 15 min, and then warmed to 70°C at a rate of 1°C/min. This temperature scheme was then cycled several times. Thermograms for IscU and IscU(D39A) were reversible with a loss of signal of ~5–10% per temperature cycle.

SAXS

Protein samples for SAXS were dialyzed extensively before data collection. Anaerobic samples were prepared by dialyzing IscU or IscU(D39A) in degassed buffer for >12 h in an anaerobic chamber (Coy Laboratories) with $O_2 < 1$ ppm and 3−6% H_2. Anaerobic samples were placed in an airtight SAXS cell within the glove box. The Bruker Nanostar benchtop SAXS system at the National Magnetic Resonance Facility at Madison was used for data collection. The Bruker Nanostar system was equipped with a rotating anode (Cu) Turbo X-Ray Source and a Vantec-2000 (2048 × 2048 pixel) detector (Bruker AXS). The sample-to-detector distance was set at ~67 cm, allowing for a detection range of $0.012\ Å^{-1} > q > 0.383\ Å^{-1}$. SAXS data were collected from IscU and IscU(D39A) samples at protein concentrations ranging from 1.5 to 6.0 mg/mL. Sample and buffer scattering data were collected over periods of 2–4 h, with frames recorded every hour. For temperature-dependent studies, the temperature was held for a minimum of 15 min before data were recorded, to allow for equilibration. The SAXS data sets were averaged and converted to 1D scattering profiles for further analysis. The ATSAS (25) software suite was used to carry out buffer subtraction and process the SAXS data. The radius of gyration (R_g) was determined by using the Guinier approximation in the q range, such that $q_{max} \leq R_g \leq 1.0$.

Minimal ensemble analysis of SAXS data

The minimal ensemble search (MES) algorithm (26) was used to carry out an ensemble analysis of temperature-dependent SAXS data from IscU. An ensemble of 1000 structures composed of fully folded to fully extended structures was used for the selection pool. Other structural models were obtained from a 100 ns molecular-dynamics trajectory generated with AMBER 12 (27), using the structured conformation of IscU as the starting structure. The simulation was carried out using a generalized Born implicit solvent model (28) along with SHAKE (29) and Langevin dynamics with a collision frequency of 2 ps^{-1} (30). A simulation temperature of 373 K was found to yield unfolded structures. The structure pool was augmented by the ensemble of 20 NMR conformers representing the solution structure of the S state of IscU (8).

We used MES to select one- and two-state ensembles that best fit the experimental SAXS data. At all temperatures, the two-state ensemble achieved a slightly better fit ($\mathcal{X}$ value) to the experimental SAXS data. Fits were also validated with the recently proposed $\mathcal{X}_{free}$ statistic. The ScÅtter software package (http://www.bioisis.net/tutorial) was used to calculate $\mathcal{X}_{free}$ with 5000 selection rounds.

RESULTS AND DISCUSSION

Under physiological conditions, IscU populates two slowly interchanging conformational states: one (S) is structured and the other (D) is dynamically disordered (Fig. 1 *A*). The S and D states interconvert with k_{ex} ~1 s^{-1} at 25°C, i.e., at a rate slow enough for separate NMR signals to be observed for the two states (31). Earlier NMR studies showed that the D state became favored at higher or lower temperatures (32), and this was confirmed more recently by circular dichroism (33). We previously determined the solution structure of the S state of IscU, which consists of four α-helices and three antiparallel β-strands (8). The D state yields a poorly dispersed ^1H-^{15}N heteronuclear single quantum coherence (HSQC) spectrum indicative of dynamic disorder (Fig. 2), and the lack of secondary chemical shifts indicates that the D state contains minimal secondary structure (34).

Prior 2D ^1H-^{15}N NMR studies of ^{15}N-labeled IscU assigned separate signals from Trp-76 (W76) $^1H^{\varepsilon1}$–$^{15}N^{\varepsilon1}$ and Lys-128 (K128) ^1H-^{15}N to the S and D states (8,35). These signals are well resolved at temperatures between 1°C and 45°C (Fig. 2), and their relative intensities in the two states as determined by FMLR (24) served as excellent probes for the relative populations of each state. This analysis of IscU

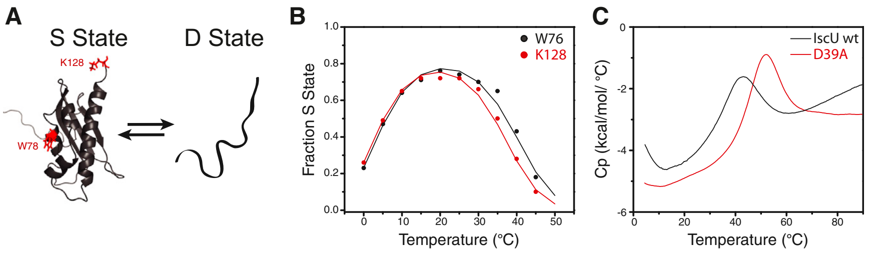

FIGURE 1 Energetic landscape of IscU. (*A*) IscU exists in equilibrium between a structured (S) state (PDB ID: 24LX) (8) and a disordered (D) state. (*B*) Temperature dependence of the fraction of the protein molecules in the S state, ([S]/([S]+[D])), as determined from the relative intensities of NMR signals assigned to W76 (*black circles*) and K128 (*red circles*) in the two conformational states. The curves resulted from fitting each data set to the Gibbs-Helmholtz equation (Table 1). (*C*) DSC thermograms of IscU (*black*) and D39A IscU (*red*).

at pH 8.0 showed that the S state is maximally populated (75%) at ~25°C, but raising or lowering the temperature shifts the equilibrium toward the D state (Figs. 1 *B* and 2). The probes located in two very different locations in the protein yielded similar temperature-dependent populations, as expected for a global conformational change (Fig. 1 *B*). The slight divergence at high temperatures likely stems from differential exchange rates of the protons with bulk water. We note that the quality of the ^{1}H-^{15}N HSQC spectra diminished with temperatures at or above 45°C, likely as the result of accelerated proton exchange with solvent. The temperature-dependent populations provided an excellent fit to the Gibbs-Helmholtz equation (Fig. 1 *B*; Table 1) (36). Thus, thermodynamically, the S-to-D transition in IscU can be described as a two-state process that is energetically symmetric at high and low temperatures.

The DSC thermogram for IscU exhibited two clear conformational transitions (Fig. 1 *C*), with upticks in the measured heat capacity near the hot and cold transition temperatures observed by NMR (Fig. 1 *B*). Cooperative transitions in proteins generally display significant heat absorption at the unfolding/melting temperature due to a sharp change in the populations of folded and unfolded states, with different enthalpies near the melting temperature (37). By contrast, noncooperative transitions, such as those observed for molten-globule states, result in linear changes in the observed partial heat capacity (Cp) as a function of temperature (38–40). Thus, the thermograms for the heat- and cold-induced transitions of IscU indicate that an extensive network of structurally stabilizing interactions is lost upon formation of the D state. The low- and high-temperature limbs are consistent with the NMR-based thermodynamic analysis, which indicated cooperative heat- and cold-induced order-to-disorder transitions with ΔH positive and negative on opposite sides of the free-energy surface. As a control, we collected DSC data on the D39A variant of IscU, whose S state is much more stable than that of wild-type IscU (8). The DSC thermogram for IscU (D39A) exhibited a single large unfolding transition at ~50°C, as expected for a classic globular protein (Fig. 1 *C*).

We next sought to gain insights into the energetic landscape of IscU by probing structural aspects of its heat- and cold-stabilized disordered conformations. A previous study showed that all four prolines of IscU have *trans* peptidyl-prolyl peptide bonds in the S state, whereas two of the four (P14 and P101) become *cis* in the heat-induced D state (41). One can determine the *cis*/*trans* conformations of peptidyl-prolyl bonds by analyzing proline side-chain chemical shifts. Specifically, the chemical-shift difference between a proline's $\delta^{13}C^{\beta}$ and $\delta^{13}C^{\gamma}$ signals is ~5 or ~10 ppm when its configuration is *trans* or *cis*, respectively (42–44). 3D HCCH TOCSY data from a sample of IscU containing [U-^{13}C]-proline at a temperature of 5°C showed the same pattern of ($\delta^{13}C^{\beta} - \delta^{13}C^{\gamma}$) chemical-shift differences observed at 45°C (41) (Fig. S1 in the Supporting Material).

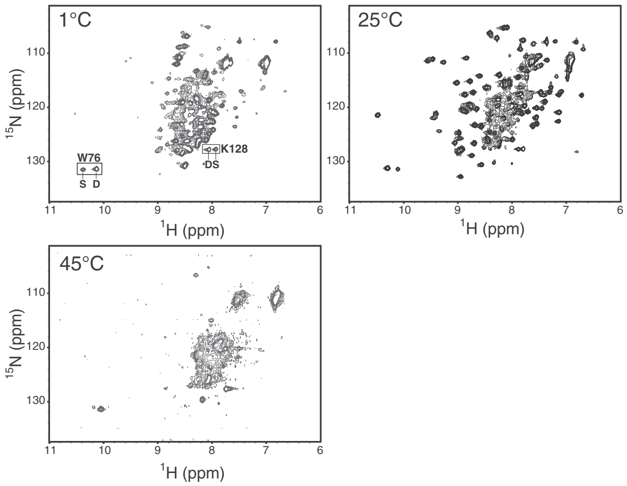

FIGURE 2 ^{1}H-^{15}N HSQC spectra of IscU collected at the temperatures indicated, showing the collapse of the spectrum upon cooling or warming. Boxes highlight the resonances used to determine the S- and D-state populations for thermodynamic analysis (Fig. 1 *B*).

TABLE 1 Fits of Temperature-Dependent NMR Data to the Gibbs-Helmholtz Equation with a Reference Temperature of 25°C

NMR Probe	ΔH (kcal · mol^{-1})	ΔS (cal · mol^{-1} · K^{-1})	ΔCp (kcal · mol^{-1} · K^{-1})
W76	6.52 ± 0.7	19.6 ± 2.3	1.71 ± 0.1
K128	10.3 ± 0.4	32.8 ± 1.4	1.82 ± 0.1

NMR data from Fig. 1 *B*. Gibbs-Helmholtz equation: $\Delta G(T) = \Delta H(T_{ref}) - T\Delta S(T_{ref}) + \Delta Cp[(T - T_{ref}) - T\ln(T/T_{ref})]$, where T_{ref} is the reference temperature.

To expand our focus from local to global structural information, we collected SAXS data, which provide low-resolution information about molecular shapes and offer a powerful means of studying disordered protein conformations (45). First, we prepared samples under buffer conditions identical to those previously used to study the structure and dynamics of IscU by NMR (8,31,34,35,41), and collected SAXS data on a Bruker Nanostar system equipped with a variable-temperature sample stage (Table S1). Surprisingly, we observed no change in the R_g of IscU upon a decrease in temperature (Fig. S2 *A*). However, as expected, the R_g of IscU increased significantly from ~23 Å to ~30 Å upon heating. To further characterize this unexpected behavior, we determined the molecular mass of IscU using the new SAXS invariant parameter, V_c (46). The V_c method has been shown to be applicable for both compact and disordered molecules. By contrast, the commonly utilized zero angle scattering $I(0)$ method (47) is most accurate when the electron density of the molecular species of interest resembles those of the molecular mass standards usually employed (globular proteins). The V_c approach yielded an average mass of 28.7 kDa over the entire temperature range (Fig. S2 *B*), a value very close to the mass of dimeric IscU (27.7 kDa). It is likely that the size of the dimeric form of IscU was masking differences between the cold- and heat-stabilized S and D states.

Subsequent SAXS studies of a protein variant, IscU (P101A), under the same buffer conditions indicated that the protein converted slowly over time from a monomer to a dimer (Fig. S3). Over the course of the variable-temperature study, the observed molecular weight shifted from that expected for a monomer to that expected for a dimer. This result led us to suspect that dimerization was occurring by formation of one or more intermolecular disulfide bridges. SAXS sample preparation requires extensive dialysis (>12 h), and the process may have led to oxidation of the reductant (DTT) in the wild-type IscU sample (Fig. S2).

To ensure that the cysteines of IscU remained reduced, we prepared samples in an anaerobic chamber. IscU was dialyzed extensively against degassed buffer containing 10 mM TCEP, and samples were placed in a sealed SAXS sample cell. IscU prepared under these conditions at 3.0 or 6.0 mg/mL appeared to be monomeric over the temperature range studied (Fig. 3). The SAXS results revealed large increases in the R_g upon cooling and heating (Fig. 3 *A*), consistent with the disorder implied from the collapsed NMR spectra at high and low temperatures.

Assuming a two-state model for the S ⇄ D equilibrium between 0°C and 50°C, the experimentally measured R_g (R_{gEXP}) is given by Eq. 1,

$$R^2_{gEXP} = p_s R^2_{gS} + p_D R^2_{gD}, \quad (1)$$

where p_S and p_D are the populations of the two states, and R_{gS} and R_{gD} are the R_g of the two states (48). We assumed

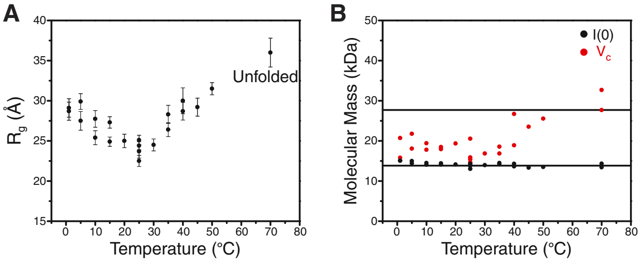

FIGURE 3 Temperature-dependent SAXS studies of monomeric IscU prepared with 10 mM TCEP in an anaerobic chamber. (*A*) R_g values calculated from SAXS data collected at different temperatures. (*B*) Molecular mass of IscU as determined from SAXS data by the $I(0)$ (*black*) and V_c (*red*) methods. The lines indicate the molecular masses of monomeric (13.8 kDa) and dimeric (27.7 kDa) IscU.

an S-state R_g of 18.9 Å over this temperature range, as derived from the NMR structural ensemble (8). As a control for the S state, we carried out temperature-dependent studies of the structured variant IscU(D39A) (8). Its R_g did not change between 0°C and 25°C, and only increased at high temperatures (Fig. S4). The calculated D-state R_g of IscU remained nearly constant (R_{gD} = 35.5 ± 2.9 Å) over the entire temperature range (Fig. 4 *A*). Interestingly, the R_g predicted for IscU in a random chain configuration is 34.2 Å (49), and this similarity to the experimental values obtained at high and low temperatures suggests that both the heat- and cold-induced D states of IscU are highly extended with very little residual structure.

We utilized an ensemble-based analysis of the SAXS data to gain deeper insight into the conformation of IscU's D state. The MES approach uses an advanced genetic algorithm to select minimal ensembles of conformers from a structure pool that best agree with experimental SAXS data (26). The conformer pool we used for MES comprised the NMR ensemble of conformers representing the S state of IscU (8) augmented by 1000 structures derived from molecular-dynamics simulations, varying from fully folded to fully extended (Fig. 4 *B*). Fig. 5 *A* shows the best fit upon MES selection for single- and two-state models. At each temperature the fit was improved by considering two conformations, and the resulting two-state fits yielded $\mathcal{X}$ and $\mathcal{X}_{free}$ values of ~1 (45). Increasing the ensemble size by up to four conformers did not result in additional improvement of the fit beyond the two-state model for all temperatures. Although the SAXS data can be fit to a single model with varying quality (~1 ≤ $\mathcal{X}_{free}$ ≤ 1.6), our NMR results clearly indicate that two states are present. This highlights the limitation of using SAXS data alone, since SAXS is unable to independently discern the difference between a single state and multiple states that satisfy the experimental IscU data. Given our knowledge from NMR that IscU exists in an S and a D state, we sought to determine what combination of the structural models (S and D) would be consistent with the SAXS data. Interestingly, when we carried out a

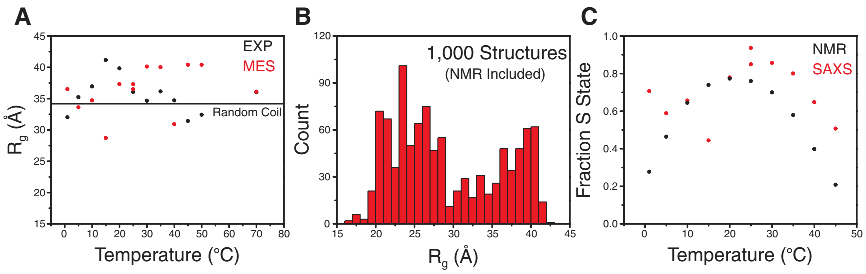

FIGURE 4 Low-resolution structural analysis of IscU by SAXS. (*A*) D-state R_g values calculated from experimental SAXS data using Eq. 1 (*black*) and from disordered structures selected from a two-state MES (*red*). (*B*) R_g values for members of the ensemble of structures used for the MES analysis. (*C*) Fractional S-state population derived from the conformers selected by the two-state MES fit (*red*) compared with that determined by NMR (*black*).

two-state MES fit, the populations of the selected S and D conformers generally trended consistently with those observed by NMR (Fig. 4 *C*). The R_g values of the disordered conformations selected by MES were also consistent with those calculated from Eq. 1 (Fig. 4 *A*). Further, the structural models selected were consistent with the NMR observation of a folded and disordered conformation (Fig. 5 *B*). Overall, our SAXS data combined with NMR suggest that the cold- and heat-induced D states of IscU are both highly extended and are nearly in a random-coil conformation. Residual structure in the D state may exist in regions around its two *cis* prolines (P14 and P101) and some secondary structure in the C-terminus.

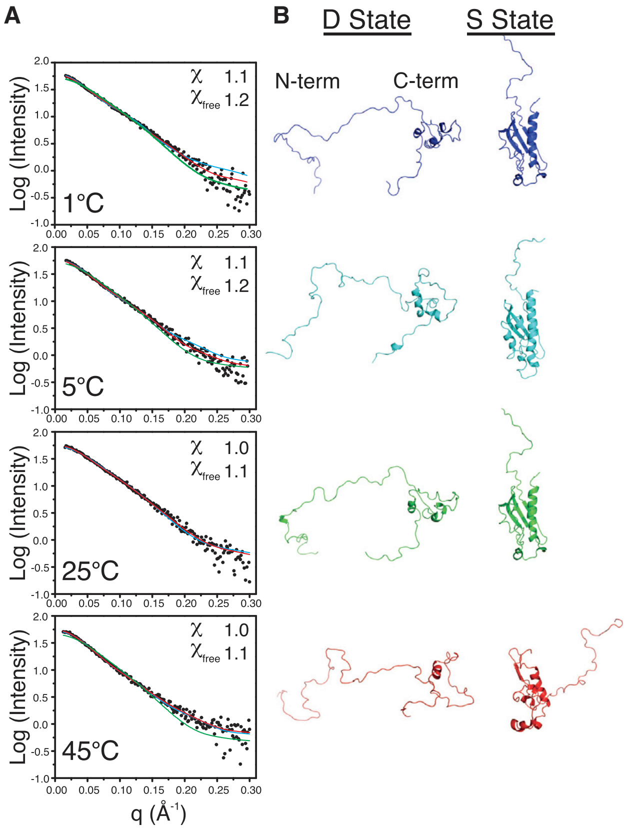

FIGURE 5 MES results for temperature-dependent IscU SAXS data. (*A*) Experimental SAXS data (*circles*) collected at different temperatures are compared with fits to MES against one structure (*blue*) and two structures (*red*). $\mathcal{X}$ and $\mathcal{X}_{free}$ statistics are shown for the two-structure MES fits. For reference, the fit (*green*) to the single structure (R_g of 22 Å) selected for IscU at 25°C is shown for 1°C, 5°C, and 45°C. (*B*) Structures selected in the two-state MES fits to the experimental SAXS data at different temperatures.

CONCLUSIONS

We utilized a hybrid approach employing NMR, DSC, and SAXS to characterize the complex energetic landscape of IscU. The low-resolution structural insight afforded by hybrid NMR/SAXS analysis suggests that the energetic landscape of IscU's S $\rightleftarrows$ D equilibrium is symmetric both energetically and structurally. Similarly to Yfh1 (12,15) and a CTL9 mutant (11,18,19), IscU underwent a two-state cooperative S→D conformational transition upon an increase or decrease in temperature. However, unlike Yfh1 and the CTL9 mutant, which exhibited more compact cold-denatured states, the heat- and cold-induced D states of IscU appeared to be equally extended. In addition, the R_g of the cold-induced D state of IscU did not increase with decreasing temperature as was observed for the cold-denatured state of Yfh1 (17). Such protein-specific differences highlight the need to develop a larger canon of model systems for studying the process of cold denaturation so that its energetic and structural aspects can be more fully understood.

SUPPORTING MATERIAL

Four figures and one table are available at http://www.biophysj.org/biophysj/supplemental/S0006-3495(15)00780-8.

AUTHOR CONTRIBUTIONS

J.L.M., J.R.B., and Z.D. designed the research. M.T., Z.D., I.K.A., and J.R.B. collected and interpreted the NMR data. J.R.B. collected and interpreted the DSC and SAXS data. R.O.F. prepared protein samples. W.M.W. carried out the molecular-dynamics simulations. All authors contributed to drafting and approving the manuscript.

ACKNOWLEDGMENTS

Financial support was provided by the National Institutes of Health (U01 GM94622). J.R.B. was supported by a Ruth L. Kirschstein postdoctoral fellowship (F32 GM110939). NMR and SAXS data were collected at the National Magnetic Resonance Facility at Madison funded by NIH grants P41 GM103399, P41 RR02301-27S1, and S10 RR027000.

SUPPORTING CITATIONS

References (8,41) appear in the Supporting Material.

REFERENCES

1. Privalov, P. L. 1990. Cold denaturation of proteins. *Crit. Rev. Biochem. Mol. Biol.* 25:281–305.
2. Wong, K. B., S. M. Freund, and A. R. Fersht. 1996. Cold denaturation of barstar: ^{1}H, ^{15}N and ^{13}C NMR assignment and characterisation of residual structure. *J. Mol. Biol.* 259:805–818.
3. Babu, C. R., V. J. Hilser, and A. J. Wand. 2004. Direct access to the cooperative substructure of proteins and the protein ensemble via cold denaturation. *Nat. Struct. Mol. Biol.* 11:352–357.
4. Pometun, M. S., R. W. Peterson, …, A. J. Wand. 2006. Cold denaturation of encapsulated ubiquitin. *J. Am. Chem. Soc.* 128:10652–10653.
5. Van Horn, W. D., A. K. Simorellis, and P. F. Flynn. 2005. Low-temperature studies of encapsulated proteins. *J. Am. Chem. Soc.* 127:13553–13560.
6. Zhang, J., X. Peng, …, J. Jonas. 1995. NMR study of the cold, heat, and pressure unfolding of ribonuclease A. *Biochemistry.* 34:8631–8641.
7. Jonas, J., L. Ballard, and D. Nash. 1998. High-resolution, high-pressure NMR studies of proteins. *Biophys. J.* 75:445–452.
8. Kim, J. H., M. Tonelli, …, J. L. Markley. 2012. Three-dimensional structure and determinants of stability of the iron-sulfur cluster scaffold protein IscU from Escherichia coli. *Biochemistry.* 51:5557–5563.
9. Kitahara, R., A. Okuno, …, K. Akasaka. 2006. Cold denaturation of ubiquitin at high pressure. *Magn. Reson. Chem.* 44:S108–S113.
10. Vajpai, N., L. Nisius, …, S. Grzesiek. 2013. High-pressure NMR reveals close similarity between cold and alcohol protein denaturation in ubiquitin. *Proc. Natl. Acad. Sci. USA.* 110:E368–E376.
11. Li, Y., B. Shan, and D. P. Raleigh. 2007. The cold denatured state is compact but expands at low temperatures: hydrodynamic properties of the cold denatured state of the C-terminal domain of L9. *J. Mol. Biol.* 368:256–262.
12. Pastore, A., S. R. Martin, …, P. A. Temussi. 2007. Unbiased cold denaturation: low- and high-temperature unfolding of yeast frataxin under physiological conditions. *J. Am. Chem. Soc.* 129:5374–5375.
13. Baez, M., C. A. M. Wilson, …, J. Babul. 2012. Expanded monomeric intermediate upon cold and heat unfolding of phosphofructokinase-2 from *Escherichia coli*. *Biophys. J.* 103:2187–2194.
14. Jaremko, M., Ł. Jaremko, …, M. Zweckstetter. 2013. Cold denaturation of a protein dimer monitored at atomic resolution. *Nat. Chem. Biol.* 9:264–270.
15. Adrover, M., V. Esposito, …, P. A. Temussi. 2010. Understanding cold denaturation: the case study of Yfh1. *J. Am. Chem. Soc.* 132:16240–16246.
16. Adrover, M., G. Martorell, …, A. Pastore. 2012. The role of hydration in protein stability: comparison of the cold and heat unfolded states of Yfh1. *J. Mol. Biol.* 417:413–424.
17. Aznauryan, M., D. Nettels, …, B. Schuler. 2013. Single-molecule spectroscopy of cold denaturation and the temperature-induced collapse of unfolded proteins. *J. Am. Chem. Soc.* 135:14040–14043.
18. Shan, B., S. McClendon, …, D. P. Raleigh. 2010. The cold denatured state of the C-terminal domain of protein L9 is compact and contains both native and non-native structure. *J. Am. Chem. Soc.* 132:4669–4677.
19. Luan, B., B. Shan, …, D. P. Raleigh. 2013. Cooperative cold denaturation: the case of the C-terminal domain of ribosomal protein L9. *Biochemistry.* 52:2402–2409.
20. Blommel, P. G., K. J. Becker, …, B. G. Fox. 2007. Enhanced bacterial protein expression during auto-induction obtained by alteration of lac repressor dosage and medium composition. *Biotechnol. Prog.* 23:585–598.
21. Kim, J. H., J. R. Bothe, …, J. L. Markley. 2014. Role of IscX in iron-sulfur cluster biogenesis in *Escherichia coli*. *J. Am. Chem. Soc.* 136:7933–7942.
22. Dai, Z., J. H. Kim, …, J. L. Markley. 2014. pH-induced conformational change of IscU at low pH correlates with protonation/deprotonation of two conserved histidine residues. *Biochemistry.* 53:5290–5297.
23. Delaglio, F., S. Grzesiek, …, A. Bax. 1995. NMRPipe: a multidimensional spectral processing system based on UNIX pipes. *J. Biomol. NMR.* 6:277–293.
24. Chylla, R. A., K. Hu, …, J. L. Markley. 2011. Deconvolution of two-dimensional NMR spectra by fast maximum likelihood reconstruction: application to quantitative metabolomics. *Anal. Chem.* 83:4871–4880.
25. Petoukhov, M. V., D. Franke, …, D. I. Svergun. 2012. New developments in the ATSAS program package for small-angle scattering data analysis. *J. Appl. Cryst.* 45:342–350.
26. Pelikan, M., G. L. Hura, and M. Hammel. 2009. Structure and flexibility within proteins as identified through small angle X-ray scattering. *Gen. Physiol. Biophys.* 28:174–189.
27. Case, D. A., T. A. Darden, …, P. A. Kollman. 2012. AMBER 12. University of California, San Francisco.
28. Nguyen, H., D. R. Roe, and C. Simmerling. 2013. Improved generalized Born solvent model parameters for protein simulations. *J. Chem. Theory Comput.* 9:2020–2034.
29. Miyamoto, S., and P. A. Kollman. 1992. Settle—an analytical version of the Shake and Rattle algorithm for rigid water models. *J. Comput. Chem.* 13:952–962.
30. Loncharich, R. J., B. R. Brooks, and R. W. Pastor. 1992. Langevin dynamics of peptides: the frictional dependence of isomerization rates of N-acetylalanyl-N′-methylamide. *Biopolymers.* 32:523–535.
31. Kim, J. H., A. K. Füzéry, …, J. L. Markley. 2009. Structure and dynamics of the iron-sulfur cluster assembly scaffold protein IscU and its interaction with the cochaperone HscB. *Biochemistry.* 48:6062–6071.
32. Markley, J. L., J. H. Kim, …, M. Tonelli. 2013. Metamorphic protein IscU alternates conformations in the course of its role as the scaffold protein for iron-sulfur cluster biosynthesis and delivery. *FEBS Lett.* 587:1172–1179.
33. Iannuzzi, C., M. Adrover, …, A. Pastore. 2014. The role of zinc in the stability of the marginally stable IscU scaffold protein. *Protein Sci.* 23:1208–1219.
34. Kim, J. H., M. Tonelli, and J. L. Markley. 2012. Disordered form of the scaffold protein IscU is the substrate for iron-sulfur cluster assembly on cysteine desulfurase. *Proc. Natl. Acad. Sci. USA.* 109:454–459.
35. Kim, J. H., M. Tonelli, …, J. L. Markley. 2012. Specialized Hsp70 chaperone (HscA) binds preferentially to the disordered form, whereas J-protein (HscB) binds preferentially to the structured form of the iron-sulfur cluster scaffold protein (IscU). *J. Biol. Chem.* 287:31406–31413.
36. Prabhu, N. V., and K. A. Sharp. 2005. Heat capacity in proteins. *Annu. Rev. Phys. Chem.* 56:521–548.
37. Privalov, P. L. 1989. Thermodynamic problems of protein structure. *Annu. Rev. Biophys. Biophys. Chem.* 18:47–69.
38. Griko, Y. V., and P. L. Privalov. 1994. Thermodynamic puzzle of apomyoglobin unfolding. *J. Mol. Biol.* 235:1318–1325.
39. Mizuguchi, M., K. Masaki, …, K. Nitta. 2000. Local and long-range interactions in the molten globule state: a study of chimeric proteins of bovine and human alpha-lactalbumin. *J. Mol. Biol.* 298:985–995.
40. Demarest, S. J., S. Deechongkit, …, P. E. Wright. 2004. Packing, specificity, and mutability at the binding interface between the p160 coactivator and CREB-binding protein. *Protein Sci.* 13:203–210.
41. Dai, Z., M. Tonelli, and J. L. Markley. 2012. Metamorphic protein IscU changes conformation by *cis-trans* isomerizations of two peptidyl-prolyl peptide bonds. *Biochemistry.* 51:9595–9602.
42. Siemion, I. Z., T. Wieland, and K. H. Pook. 1975. Influence of the distance of the proline carbonyl from the beta and gamma carbon on the ^{13}C chemical shifts. *Angew. Chem. Int.Engl.* 14:702–703.
43. Shen, Y., and A. Bax. 2010. Prediction of Xaa-Pro peptide bond conformation from sequence and chemical shifts. *J. Biomol. NMR.* 46:199–204.

44. Schubert, M., D. Labudde, …, P. Schmieder. 2002. A software tool for the prediction of Xaa-Pro peptide bond conformations in proteins based on ^{13}C chemical shift statistics. *J. Biomol. NMR.* 24:149–154.

45. Putnam, C. D., M. Hammel, …, J. A. Tainer. 2007. X-ray solution scattering (SAXS) combined with crystallography and computation: defining accurate macromolecular structures, conformations and assemblies in solution. *Q. Rev. Biophys.* 40:191–285.

46. Rambo, R. P., and J. A. Tainer. 2013. Accurate assessment of mass, models and resolution by small-angle scattering. *Nature.* 496:477–481.

47. Rambo, R. P., and J. A. Tainer. 2011. Characterizing flexible and intrinsically unstructured biological macromolecules by SAS using the Porod-Debye law. *Biopolymers.* 95:559–571.

48. Choy, W. Y., F. A. Mulder, …, L. E. Kay. 2002. Distribution of molecular size within an unfolded state ensemble using small-angle X-ray scattering and pulse field gradient NMR techniques. *J. Mol. Biol.* 316:101–112.

49. Fitzkee, N. C., and G. D. Rose. 2004. Reassessing random-coil statistics in unfolded proteins. *Proc. Natl. Acad. Sci. USA.* 101:12497–12502.

Complex energy landscape of the protein IscU

Jameson R. Bothe[a,b], Marco Tonelli[b], Ibrahim Ali[a], Ziqi Dai[a,b], Ronnie O. Frederick[c], William M. Westler[b], and John L. Markley[a,b,c]*

[a]Department of Biochemistry, [b]National Magnetic Resonance Facility at Madison, and [c]Mitochondrial Protein Partnership, Center for Eukaryotic Structural Genomics University of Wisconsin, 433 Babcock Drive, Madison, WI 53706

*To whom correspondence should be addressed.

Phone: (608) 263-9349. Fax: (608) 262-3759.

Email: jmarkley@wisc.edu.

Supporting Material

TABLE S1. Summary of the SAXS data.

Sample	IscU
Data-collection parameters	
Instrument	Bruker Nanostar
Beam geometry	2 pinholes (500 μm)
Wavelength (Å)	1.5418 Å
q range ($Å^{-1}$)	0.012 – 0.384
Exposure time (h)	2-4
Concentration range ($mgml^{-1}$)	1.5-6.0
Temperature (K)	274 - 343
Molecular-mass determination	
Molecular mass [I(0)] (kDa)	Sample Prep Dependent
Molecular mass [V_c] (kDa)	Sample Prep Dependent
Calculated molecular mass from sequence (kDa)	13.9
Software Employed	
Primary data reduction	SAXS (Bruker)
Data processing	PRIMUS
Computation of model intensities	FoXS
Ensemble fitting	MES
Three-dimensional graphics representations	Pymol

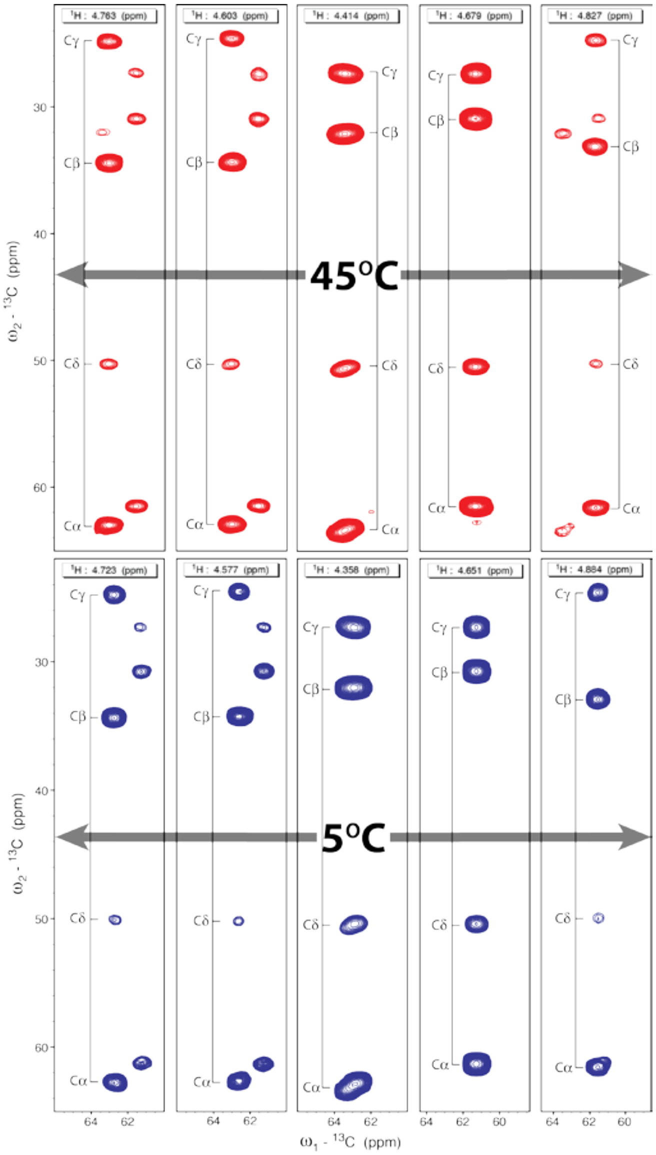

FIGURE S1 Slices through 3D HCCH-TOSCY spectra at the ^{1}H frequencies shown at the top of each slice. Proline residues of IscU in both its heat- and cold-induced disordered states adopt a similar mixture of *cis* and *trans* peptide bonds as exhibited by the chemical shift difference between the $^{13}C^{\beta}$ and $^{13}C^{\gamma}$ signals. Strips 1, 2, and 5 have ($\delta^{13}C^{\beta} - \delta^{13}C^{\gamma}$) ≈ 10 ppm indicative of a *cis* peptidyl prolyl peptide bond, and strips 3 and 4 have ($\delta^{13}C^{\beta} - \delta^{13}C^{\gamma}$) ≈ 5 ppm indicative of a *trans* peptidyl prolyl peptide bond. The strips at 45 °C were assigned previously (1) as follows: strips 1 and 2 to two conformations of Pro14; strip 3 to Pro 35, strip 4 to Pro100, and strip 5 to Pro101.

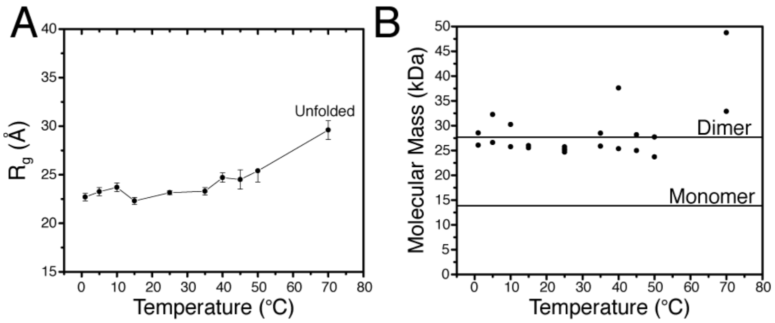

FIGURE S2 Temperature-dependent SAXS studies of IscU prepared by dialyzing into a buffer with 5 mM DTT. (A) Radius of gyration (R_g) calculated from SAXS data collected at different temperatures. (B) Molecular mass of IscU determined from the SAXS data by the V_c method. The lines indicate the molecular masses of monomeric (13.8 kDa) and dimeric (27.7 kDa) IscU.

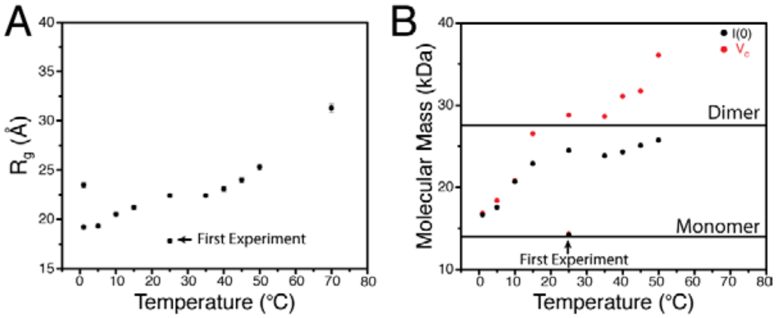

FIGURE S3 Evidence for the dimerization of IscU over time as DTT oxidizes. (A) Radius of gyration (R_g) calculated from SAXS data collected at different temperatures. The first experiment was recorded at 25 °C. The sample was then cooled to 1°C and the temperature was raised in steps to 70 °C. (B) Molecular mass of IscU determined from SAXS data by the *I*(0) (red) and V_c (black) methods. The lines indicate the molecular masses of monomeric (13.8 kDa) and dimeric (27.7 kDa) IscU.

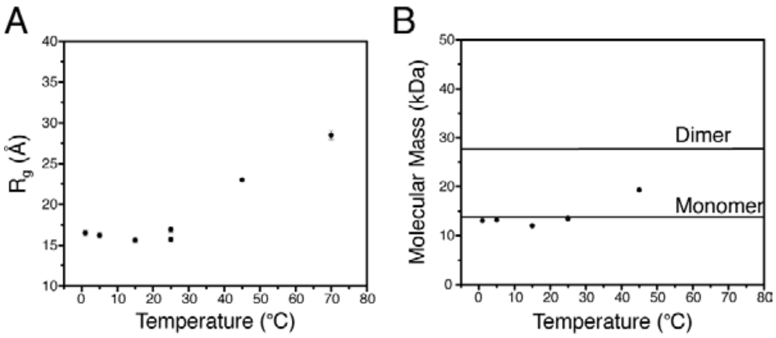

FIGURE S4 Temperature-dependent SAXS data for IscU(D39A), which has a much more stable S-state than wild-type IscU (2). (A) experimentally observed R_g. (B) molecular mass of IscU(D39A) determined by the V_c method. The lines indicate the molecular masses of monomeric (13.8 kDa) and dimeric (27.7 kDa) IscU(D39A). Unlike IscU, the R_g of IscU(D39A) does not increase significantly upon decreasing the temperature. From NMR studies, the S-state of IscU(D39A) is populated at ~95%. IscU(D39A) transitions to the D-state at 50 °C and unfolds completely at 70 °C.

References

1. Dai, Z., M. Tonelli, and J. L. Markley. 2012. Metamorphic Protein IscU Changes Conformation by *cis-trans* Isomerizations of Two Peptidyl-Prolyl Peptide Bonds. *Biochemistry* 51:9595-9602.

2. Kim, J. H., M. Tonelli, T. Kim, and J. L. Markley. 2012. Three-Dimensional Structure and Determinants of Stability of the Iron-Sulfur Cluster Scaffold Protein IscU from *Escherichia coli*. *Biochemistry* 51:5557-5563.
